# Possible Roles for Purinergic Receptor P2RX4 in Breast and Prostate Cancers

**DOI:** 10.3390/ijms26189043

**Published:** 2025-09-17

**Authors:** Walfred H. J. Ho, Kong-Peng Lam

**Affiliations:** 1 School of Physical and Mathematical Sciences, Nanyang Technological University, Singapore 637371, Singapore; hohs0003@e.ntu.edu.sg; 2 Singapore Immunology Network (SIgN), Agency for Science, Technology and Research (A*STAR), 8A Biomedical Grove, #04-06 Immunos, Singapore 138648, Singapore; 3 Department of Microbiology and Immunology, Yong Loo Lin School of Medicine, National University of Singapore, Singapore 117593, Singapore; 4 School of Biological Sciences, Nanyang Technological University, 60 Nanyang Drive, Singapore 637551, Singapore

**Keywords:** purinergic signaling, tumor, autophagy, lysosomes

## Abstract

The purinergic receptor P2RX4 contributes to the malignant behavior of breast and prostate cancers. It is upregulated in these malignancies and promotes tumor progression through mechanisms involving EMT, autophagy, and the release of pro-malignant lysosomal contents. P2RX4 also influences cellular signaling pathways by interacting with oncogenes and tumor suppressors. Certain genetic variants of P2RX4 are associated with an increased risk of developing these cancers. Hence, targeting P2RX4 represents a promising therapeutic approach, with potential strategies including antibody and CAR-T cell therapies and the development of small-molecule inhibitors. Further investigation is needed to fully elucidate the molecular mechanisms by which P2RX4 drives cancer progression and to translate these findings into effective and safe clinical therapies.

## 1. Introduction

Cancer is a leading cause of death globally and is associated with abnormal and uncontrolled cell growth and the spread of malignant cells to healthy tissues. One in six deaths worldwide is caused by cancer [1], and this phenomenon has led to many studies into this complex disease. The complexity of cancer lies in both the causes and mechanisms underlying oncogenesis and disease progression. By understanding the etiology of cancers, we can find better ways to manage and treat these ailments.

From a study point of view, healthy and cancerous cells can be sequenced to identify their genetic defects, particularly in oncogenes or tumor suppressor genes [2]. Oncogenes are genes that have the potential to cause cancer through gain-of-function mutations or overexpression. Tumor suppressor genes, such as p53, which regulates cell replication [2,3], lose their normal functions when mutated, and the mutated variant contributes to the etiology and/or progression of cancer. Hence, the identification of genetic aberrations in oncogenes and tumor suppressor genes could contribute to better understanding and treatment of cancer.

Though not considered as oncogenes or tumor suppressor genes, the P2X family of receptors has been shown to play physiologically important roles in cell signaling [4] and could contribute to the progression of cancer. P2RX4, a member of this family, has been implicated in breast and prostate cancers [5,6,7] and shown to promote tumor progression and aggressiveness [7]. Because of the worldwide prevalence of breast and prostate cancers [1] and their increasing occurrence in the Asian population [8,9], we review here the role of P2RX4 and its signaling pathways that could be involved in cell physiological and oncogenic processes.

## 2. Purinergic Receptors

**Figure 1:**
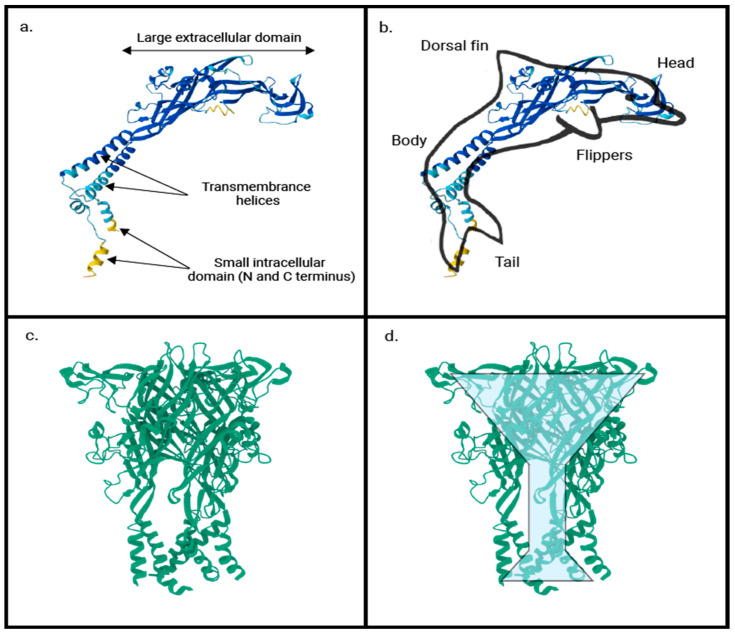
Schematic depiction of P2X receptor structure showing (**a**) monomeric 3D structure, (**b**) imposed upon a picture of a dolphin, (**c**) trimeric 3D structure and (**d**) superimposed on a chalice. Structures extracted from protein database [16] (4dw0) and modified from Ref. [13].

To better understand P2RX4, we first review the broader family of P2X receptors and purinergic signaling and their roles in various physiological processes. Purinergic signaling is mediated by 7 subtypes of purinergic receptors of the P2X (also known as P2RX) family. P2X receptors (P2RX1–7) are 6-pass transmembrane ion channels activated by ATP, the universal currency of cellular energy [10]. They have different tissue distribution patterns and are activated by different concentrations of ATP [11,12]. P2X receptors functionally co-assemble as trimers with each subunit shaped like a dolphin, and the trimeric functional receptor shaped like a chalice [13] (Figure 1). Co-assembly of functional receptors can be with the same or different subunits. However, the physiological significance of the various heterotrimeric receptors has yet to be elucidated. These functional receptors are surface-bound ion channels permeable to Na^+^, K^+^ and Ca^2+^ ions [11,14]. P2X receptors play a role in transporting ions that are needed for physiological processes such as muscle contraction and neurotransmission. For example, Ca^2+^ activates calmodulin, leading to muscle contraction [14], while the transport of Na^+^ and Ca^2+^ ions across membrane leads to depolarization during neurotransmission [15].

P2X receptors are widely expressed in various tissues and cells such as the brain, muscle, epithelial, endothelial and immune cells. One or more P2X receptor subtypes can be expressed on the same cell type. Most P2X receptors are found on neurons and muscle cells. We summarized the distribution of P2X receptors in Table 1.

## 3. P2X Receptors and Their Roles in Disease and Cancer

ATP is the currency of cell energetics and can regulate the functions of P2X receptors. At physiological states, ATP is mostly absent in the extracellular matrix (ECM). ATP-regulated cell processes are tightly controlled and terminated quickly due to the actions of surface membrane proteins such as CD23 and CD79 that cleaves extracellular ATP (eATP) to adenosine, which is then taken up by cells to be converted into ATP for intracellular storage [21,22]. Elevated eATP concentrations occur when cells release ATP in response to stress or damage. Non-programmed cell death (necrosis) also releases ATP into the ECM [23,24]. A much higher level of eATP concentration was also observed in the tumor microenvironment [22].

ATP in pathological environments can activate P2X receptors, leading to inflammatory signaling and the release of cytokines and pro-inflammatory molecules [23]. Inflammation then drives the release of more ATP from nearby cells, which propagates the inflammatory processes by the activation of P2X receptors on both immune and non-immune cells [25]. Inflammatory signaling pathways can exacerbate disease conditions in patients. Defective expression of P2X receptors can also contribute to diseases involving neurotransmission, muscle contraction and organ or tissue functions [25,26,27,28,29,30,31,32,33,34,35,36,37,38,39,40,41,42,43]. We summarize the involvement of P2X receptors in diseases in Table 2.

In the past few years, there have been studies showing the involvement of ion channels in cell growth, proliferation and differentiation, especially in oncogenesis. Ion channels expressed on cancer cells are potential targets for cancer treatment, such as the L-type voltage-gated ion channels [44]. P2X receptors are no exception due to their expression on many tumor cells, and certain P2X receptors are overexpressed in some cancers [4,6,45]. Unusually high ATP concentrations in the tumor microenvironment (TME) can activate P2X receptors on cancer cells, leading to their increased proliferation and metastasis [46,47]. Because of such pro-oncogenic roles, P2X receptors are generally viewed as good targets for cancer treatment [46,47,48].

P2RX7 is one of the most studied P2X receptors due to its role as a mediator of ATP-dependent apoptotic or necrotic cell death [49,50]. The current focus of P2RX7 therapeutics seems to gravitate towards both chronic inflammatory diseases and cancer [5,51]. Unfortunately, phase 2b clinical trials involving P2RX7-targeting rheumatological drugs have yielded inconclusive results [52]. On a brighter note, a promising anti-P2RX7 mAb treatment against Basal Cell Carcinoma (BCC) passed phase 1 clinical trials [53]. The trial results showed that 65% of the patients responded to this mAb treatment with significant reduction in BCC lesion size, indicating the potential for P2X-targeting mAb drugs for cancer therapy.

Other P2X receptors have also been gaining attention for the development of cancer therapeutics due to their overexpression in cancers. For instance, P2RX4 is overexpressed in leukemia [54], breast [55], prostate [7,56], and liver cancers [45]. Similarly, P2RX5 is overexpressed in lymphoid malignancies such as T-cell leukemia and multiple myeloma [38]. The overexpression of P2X receptors in these cancers renders them attractive targets for developing novel cancer therapeutics or biomarkers.

## 4. P2RX4: A Dual-Function Purinergic Receptor

P2RX4 stands out as the most unique of the P2X family of receptors. It has the widest expression and is found on epithelial, endothelial, neuronal, skeletal muscle and immune cells [57,58]. P2RX4 is also the most sensitive to ATP concentration and has one of the highest permeability to calcium ions [57] (Ca^2+^). P2RX4 exhibits dual roles at the plasma membrane and in the lysosomes. P2RX4 is constantly transported between the plasma membrane and the endosomes and is mostly localized to the lysosomes [57,59,60]. Once activated by ATP, they undergo endocytosis to the lysosomes and can be trafficked to the plasma membrane during exocytosis events [59,60].

ATP binding desensitizes the functional receptor and P2RX4 is trafficked to the lysosomes where the bound ATP detaches, and the process enables P2RX4 recycling. The acidity of the lysosomes resensitizes the P2RX4 receptor through protonation of histidine residues [61], where it can be recycled back to the plasma membrane to resume its function [62]. Because of N-glycosylation, P2RX4 is protected from lysosomal degradation and is able to function under the harsh luminal environments of the lysosomes [63], where it plays the second role of regulating lysosomal fusion and ion concentrations, which are required for lysosomal enzyme activity [64] as well as exocytosis and post-membrane fusion events [65].

**Figure 2:**
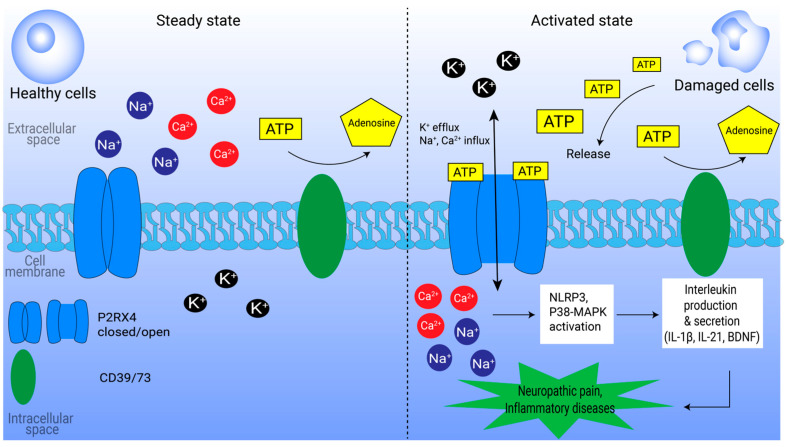
Involvement of P2RX4 in inflammation and neuropathic pain. At steady state (**left Panel**), ATP concentration in the extracellular matrix is negligible and quickly degraded by CD39/73. Upon cell damage (**right Panel**), ATP is released into the extracellular matrix, and the higher ATP concentrations activate P2RX4 on neighboring cells. Activated P2RX4 allows the transport of important ions such as calcium and potassium into the cell, which activates the inflammasomes such as NLRP3 and signaling pathways such as P38-MAPK, triggering the production of molecules such as interleukins which will exacerbate inflammation and pain.

The localization of P2RX4 to the plasma membranes is also physiologically important. Firstly, ATP binds tightly to P2RX4 subunits to activate the ion channel, allowing non-specific ions such as K^+^ to exit and Ca^2+^ and Na^+^ to enter [66], which can subsequently activate downstream pathways and molecules such as the inflammasomes, leading to neuropathic pain and inflammatory diseases [67] (Figure 2). Other than a promising target for inflammatory diseases, P2RX4 has potential to be a good cancer target but was mostly overlooked until recently [6,46].

## 5. P2RX4 in Breast Cancer Aggressiveness, Epithelial–Mesenchymal Transition & Autophagy

Breast cancer is currently one of the most prevalently diagnosed cancers affecting women with an estimated number of 2.3 million new cases worldwide and is the 5th cause of cancer-related deaths [1]. Though early-stage breast cancers can be easily treated using current medical strategies, the more advanced cases have proven to be more challenging [68]. Currently, challenges in cancer treatment are caused by the ability of cancer cells to transform to a more invasive phenotype and their acquisition of new abilities to fuel their high metabolism rates for survival in the nutrient-deprived and hypoxic tumor microenvironment [68,69].

The ability of breast cancer cells to undergo Epithelial–Mesenchymal Transition (EMT) contributes to its aggressiveness and invasiveness. EMT causes the typically polarized epithelial cells to lose their polarity and gain the ability to enter circulation [70]. Another ability of cancer cells is their enhanced autophagy involving acidic lysosomes digesting cellular components to provide energy for the tumor cells under stressful and nutrient-sparse conditions [71]. In breast cancer, EMT and autophagy are promoted by P2RX4 as observed by changes in the transcriptional expression of mesenchymal markers and autophagy markers p62 when P2RX4 is silenced [55]. It was observed that silencing of P2RX4 leads to downregulation of mesenchymal markers Vim and Twist as well as the upregulation of epithelial markers Zo1 and Cdh1, reversing EMT and reducing tumor cell invasion [55].

**Figure 3:**
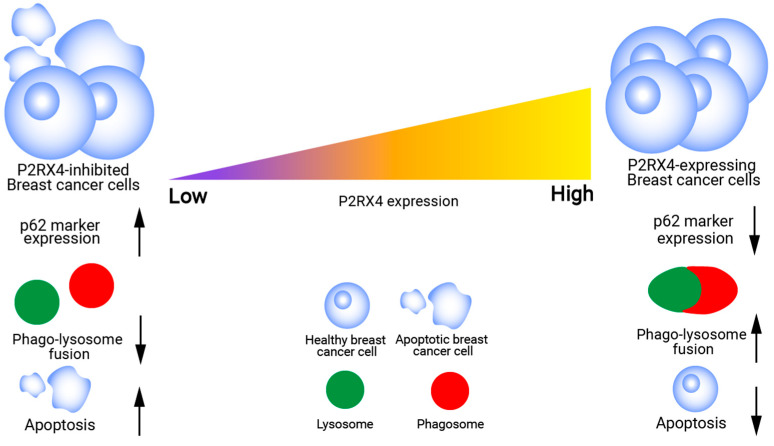
Involvement of P2RX4 Relationship between P2RX4 expression, autophagic activity and breast cancer cell survival, as reported previously [55]. Inhibition of P2RX4 led to reduced lysosome fusion with phagosomes, diminished autophagic flux as indicated by increased p62 expression, and increased cell death.

P2RX4 signaling also affects autophagy, which could increase the aggressiveness of breast cancer cells (Figure 3). Autophagy allows cancer cells to thrive in nutrient-deprived environments where healthy cells would normally die [72]. This process serves two purposes: one for protecting cells from toxins and the other for maintaining high cell metabolism and energy balance to support cell survival [71,72]. Studies have shown that autophagic activities are upregulated in breast cancer [73,74,75]. Inhibition of P2RX4 by 5-BDBD (P2RX4-specific small-molecule antagonist) significantly increased p62 marker expression, which is indicative of autophagic impairment [55]. Increased apoptosis and greater amounts of unfused lysosomes were also observed in cells during P2RX4 silencing [55,76]. Reversal of P2RX4 knockdown significantly decreased p62 levels, and increase autophagy flux and lysosomal fusion [55], thus enhancing tumor cells’ aggressive capabilities. Targeting autophagic abilities in breast cancer to stem their aggressiveness has been suggested by previous studies [73,76,77], and P2RX4 could be a good target for impairing autophagy for breast cancer therapy.

## 6. P2RX4-Mediated Lysosomal Exocytosis in Breast Cancer

Lysosomal exocytosis, a membrane-fusion process, which results in the expulsion of soluble debris after autophagic digestion, serves as a waste clearance mechanism [78,79]. In breast cancer, the expelled contents could modulate cancer cell survival and invasion [78,79]. Cancer cells frequently employ lysosomal exocytosis to break down ECM components and tissue barriers, which would in turn promote tissue invasion and metastasis [79,80,81]. Lysosomal proteins, specifically proteases when released into the ECM can remodel it to promote tumor growth and invasion [79,80,81]. Cathepsin D (CTSD) has the ability to break down ECM and enables cancer extravasation which has been widely linked to cancer severity and adverse survival outcomes for patients [82,83]. In breast cancer, CTSD and P2RX4 expression are correlated, where tumor and metastatic cells have much higher levels of CTSD in the ECM and P2RX4 expression compared to healthy neighboring cells [55]. In addition, growth factors and invasive signaling molecules (EGF, TGF) can be released via lysosomal exocytosis and received by neighboring tumor cells [84,85,86].

**Figure 4:**
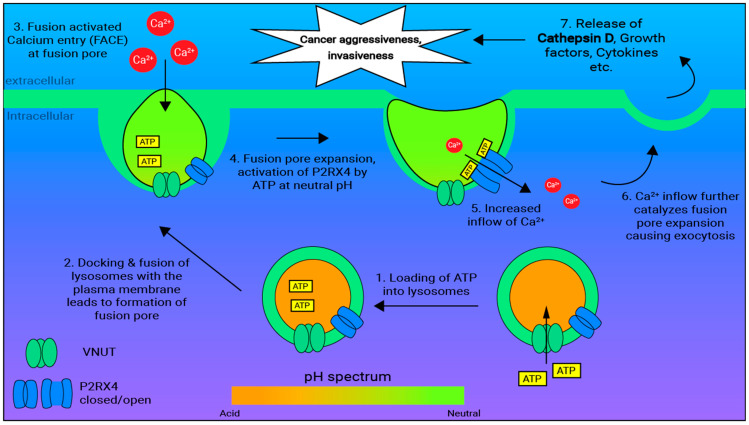
P2RX4 facilitates lysosomal exocytosis in breast cancer by promoting FACE. Lysosomal exocytosis is dependent on the fusion of the lysosome with the plasma membrane, which involves VNUT and P2RX4. VNUT is responsible for loading ATP into the lysosomal lumen which is needed for P2RX4 activation. P2RX4 is only activated by the loaded ATP when the lysosome pH is neutralized during initial docking and pore formation at the plasma membrane. P2RX4 promotes fusion pore expansion which drives exocytosis via a process known as FACE, which involves inflow of Ca^2+^ into the cell. Localized increased concentration of Ca^2+^ further drives the expansion of the pore and the release of contents into the extracellular matrix.

Lysosomal exocytosis is a calcium-mediated process, which can be regulated by P2RX4 (Figure 4). Anterograde transport of lysosomes towards the plasma membrane activates Vascular Nucleotide Transporter (VNUT) to load cytosolic ATP into the lysosomal lumen, but the acidity of the lysosomes prevents the premature activation of P2RX4 [87]. Initial fusion of the lysosomes with the plasma membrane creates a pore between the extracellular domain and lysosomal lumen [60,84,88]. During pore formation, the acidic pH in the lysosomes is neutralized, and ATP in the lysosomal lumen activates P2RX4, causing a spike in calcium ions flowing from the pore into the cytosol [60,89].

The localized spike in calcium concentration signals lysosomes docking and fusion near the plasma membrane in a process known as ‘Fusion-Activated Ca^2+^-Entry’ (FACE). FACE promotes pore expansion and the release of growth factors and CTSD into the ECM [84]. Increased P2RX4 expression was observed to also promote lysosomal exocytosis [55], allowing higher concentrations of CTSD to be released into the extracellular matrix, promoting degradation of the ECM and enhancing the invasive properties of cancer cells.

## 7. P2RX4 and Prostate Cancer Aggressiveness and Tumor Growth

Prostate cancer (PCa) is one of the most frequently diagnosed cancers in men. Like breast cancer, early intervention allows an almost 100% survival rate for patients with localized tumors, but complications arise in more advanced cases [1,90]. There is an increasing detection of prostate cancer amongst Asian men facilitated by prostate cancer screening and improvement in biopsy methods [8,90,91,92]. Unfortunately, there is also increasing poor prognosis for PCa in Asian men. Compared to migratory Asians and westerners, men in developed Asian countries such as Singapore have a higher proportion of metastatic PC [8,91].

Studies have found that among the seven P2X receptor family members, P2RX4 is the highest expressed functional P2X receptor in PCa [56,93]. Similar to breast cancer, P2RX4 is expressed significantly higher in PCa patient tissues and cell lines (PC3, LNCap, C4-2B4) compared to benign and healthy tissues [56,93]. In PCa, P2RX4 is mostly expressed in prostate-localized immune cells and prostate epithelial cells [56].

P2RX4 mediates tumor growth and invasiveness in prostate cancer [56,93]. Studies in mouse models using P2RX4-specific inhibitors such as 5-BDBD and PSB-12062 demonstrated a 50% reduction in the invasive capabilities of PCa [93]. Inhibition of P2RX4 did not cause any direct cell death but stunted the growth rate of PCa tumor cells in mouse xenograft model by 39.4% in 2 weeks while in cell culture model, growth rates were reduced by 50–70% [93].

Like breast cancer, P2RX4 also promotes EMT in PCa cells [56,94]. Activation of P2RX4 on PCa cells by ATP or CTP leads to decreased expression of epithelial markers such as E-cadherin and cytokeratin 8, and increased expression of mesenchymal markers such as snail and vimentin [56,94]. By silencing P2RX4, the EMT phenomenon in PCa cells can be reversed, and cells adopt their native epithelial phenotype [56,94].

## 8. P2RX4 Modulates Oncogenes and Tumor Suppressor Genes in Prostate Cancer

PTEN, a tumor suppressor gene encoding a phosphatase, is commonly inactivated and linked to more aggressive outcome in PCa. Interestingly, P2RX4 is expressed significantly higher in PTEN-inactivated PCa tissues compared to PTEN-expressing PCa tissues [56,94,95]. It was also shown that PTEN-expressing PCa has reduced invasive capabilities compared to PTEN-inactivated PCa. The expression of ERG, an ETS-related gene linked to aggressive PCa and acts as a transcription factor promoting cell proliferation and angiogenesis in PCa, correlates with P2RX4 expression [56,95,96]. ERG-positive PCa associated cells have much higher expression of P2RX4 than ERG-negative PCa cells [56]. Though the pattern of P2RX4-PTEN and P2RX4-ERG expression have been characterized in PCa, the molecular mechanism behind such an observation remains unknown.

Exposing PCa cells to TGFβ-1 induces EMT [97] through the activation of the PI3 kinase/Akt pathway and upregulates the oncogene Akt, which is a mediator of PCa EMT [98]. The phosphatases PTEN and PHLLP regulate Akt and are shown to engage in crosstalk, influencing EMT [94,98]. This crosstalk was found to be specific to TGFβ-1-induced PCa cells where PTEN and PHLPP are downregulated in a reciprocal manner, to control Akt levels to promote PCa metastasis [56,94]. Overexpression of one phosphatase leads to the downregulation of the other. It was found that P2RX4, through its role in Calcium signaling [61,94] mediates this PTEN-PHLLP crosstalk [94]. Silencing P2RX4 interferes with this crosstalk and TGFβ-1-induced EMT was prevented and key PCa markers linked with cancer aggressiveness such as MMP9 were downregulated [94]. These studies suggested that PTEN-loss was associated with higher P2RX4 expression, which could modulate other oncogenes and enhance the invasiveness of PCa cells.

## 9. P2RX4 Variants and Risk of Breast and Prostate Cancer

P2RX4 polymorphism has been linked to the development of various cancers. The P2RX4 homozygous gene variant rs25644:G/G is associated with a high risk of prostate cancer [99]. Compared to the heterozygous gene variant rs25644:G/A, subjects carrying the homozygous variant have 4.79 times higher expression of P2RX4 [99]. As covered previously, higher P2RX4 expression in the prostate is linked to higher risk of developing PCa, but the mechanistic role of this polymorphic P2RX4 in the development of PCa is still lacking.

The P2RX4 genotypes GA/GG in rs28360472 were associated with an increased risk of breast cancer, mesothelial and soft tissue cancers. This variant causes a mutation from tyrosine to cysteine at position 315 supposedly causing a ‘loss of function’ by not binding ATP [99,100]. It could be possible that this mutation could lead to the activation of other signaling pathways or change of function of P2RX4 that could accelerate oncogenesis.

## 10. P2X-Related Cancer Therapeutics

Currently, there is no P2RX4-targeting drug on the market for cancer therapy. Small molecules used in studies such as PSB-12062 or 5-BDBD remain solely for experimental purposes [61,101]. Monoclonal antibodies developed against P2RX4 are also mostly used for analytical studies and are raised against mouse or rat P2RX4 [101]. However, these antibodies generated by past studies help to shed light on the use of P2RX4-related antibody as therapeutics.

Antibody and cell-based therapies show great promises in targeting P2X4 for cancer treatment. As anti-P2RX7 monoclonal antibodies (mAbs) have demonstrated efficacy in clinical trials for BCC [53], it is not unreasonable to attempt to engineer anti-P2RX4 monoclonal therapeutic antibodies with inhibitory abilities to block its downstream signaling pathways. Potential anti-P2RX4 mAbs developed against the head domain have the potential to inhibit channel function and relieve neuropathic pain in mouse models [102].

In addition, conjugation of anti-P2RX4 Ab with cytotoxic drugs (ADCs) or oligonucleotides (AOCs) can deliver anti-cancer drug payloads [103,104,105], which could selectively and potently target and kill cancer cells that overexpress P2RX4. Similarly, engineering of bispecific antibodies that concurrently bind P2RX4 and CD3 [106] could recruit and signal T cells to attack cancer cells that overexpress P2RX4.

CAR-T cell therapy can offer another powerful approach to target P2RX4-expressing cancers. T cells from patients can be harvested and engineered to express a chimeric antigen receptor (CAR) specific to P2RX4 to effectively recognize and eliminate cancer cells overexpressing P2RX4 [107]. CAR-T therapy has been used in hematological malignancies of B-cells and multiple myeloma [108,109]. A recent study demonstrated the dependence of plasma cells on P2RX4 for survival [110] and CAR-T can be harnessed to control pathogenic plasma cells via P2RX4 targeting.

Immunotherapeutic strategies hold potential for the treatment of breast and prostate cancers that overexpress P2RX4, and further research is needed to optimize their efficacy and minimize side effects. Rigorous preclinical and clinical studies are essential to evaluate the safety and efficacy of these therapies given the expression pattern of P2RX4. By developing targeted therapies against P2RX4, we may be able to improve patient outcomes and combat the devastating effects of aggressive cancers as shown in the case of targeting P2RX4 in pathogenic plasma cells [110].

## 11. Concluding Thoughts

In this review, we highlighted the role of the purinergic receptor P2RX4 in promoting tumor aggressiveness, particularly in breast and prostate cancers. Upregulation of P2RX4 has been linked to increased tumor cell proliferation, migration, and invasion through several processes such as the promotion of EMT, autophagy and lysosomal exocytosis, as well as in the modulation of oncogene and tumor suppressor gene crosstalk via unelucidated mechanisms. We also showcased some P2RX4 polymorphisms that could be linked to an increased risk of cancer.

Targeting P2RX4 presents a promising therapeutic approach for combating some of these aggressive cancers. Future studies should delve deeper into the molecular mechanisms underlying P2RX4-mediated cancer progression, such as the role of P2RX4 in PTEN-PHLLP crosstalk, and to engineer novel anti-P2RX4 therapeutics with improved efficacy and selectivity. Potential immunotherapies such as CAR-T, mAbs or ADCs should be developed to specifically target cancer cells while avoiding normal tissues to reduce undesired toxicities. By advancing our understanding of P2X4 and developing highly specific therapies, we hope to improve patient treatments and outcomes in cases of more aggressive cancers.

## Figures and Tables

**Table 1:** General tissue distribution of P2X receptors.

P2X Receptor	Tissue Distribution	References
P2RX1	Smooth muscle, platelets, cerebellum, dorsal horn spinal neurons	[11,12,14,17,18]
P2RX2	Smooth muscle, CNS, retina, chromaffin cells, autonomic and sensory ganglia	[11,12,15,17,18,19,20]
P2RX3	Sensory neurons	[11,12,15,17,18,19,20]
P2RX4	Central nervous system, epithelial cells, endothelial cells, cardiac myocytes, immune cells	[11,12,15,17,18,20]
P2RX5	Skeletal muscle cells, bone neurons and epithelial cells	[11,12,17]
P2RX6	Central nervous system	[11,12,17,19]
P2RX7	Immune cells, central nervous system	[11,12,15,17,18,20]

**Table 2:** P2X receptors and their involvement in diseases.

P2X Receptor	Disease Implication	References
P2RX1	Impaired kidney function, male infertility	[26,27,28]
P2RX2	Bladder hyporeflexia, impaired taste sensing	[29,30,31]
P2RX3	Reduce pain sensing, impaired gut peristalsis	[32,33,34]
P2RX4	Inflammatory diseases, neuropathic pain	[35,36,37]
P2RX5	Lymphoid malignancies, bone loss	[38,39]
P2RX6	Chronic heart diseases	[40]
P2RX7	Inflammatory diseases, neurodegenerative diseases	[41,42,43]
